# Dynamic Shear Modulus and Damping Ratio of Sand–Rubber Mixtures under Large Strain Range

**DOI:** 10.3390/ma13184017

**Published:** 2020-09-10

**Authors:** Jianfeng Li, Jie Cui, Yi Shan, Yadong Li, Bo Ju

**Affiliations:** 1 School of Civil Engineering, Guangzhou University, Guangzhou 510006, China; 2111816143@e.gzhu.edu.cn (J.L.); jcui2009@hotmail.com (J.C.); shanyi317403389@gmail.com (Y.S.); jubo2019@163.com (B.J.); 2 Guangdong Engineering Research Center for Underground Infrastructural Protection in Coastal Clay Area, Guangzhou 510006, China

**Keywords:** sand–rubber mixture, cyclic triaxial testing, dynamic shear modulus, damping ratio, empirical model

## Abstract

Adding rubber into sands has been found to improve the mechanical behavior of sands, including their dynamic properties. However, ambiguous and even contradictory results have been reported regarding the dynamic behavior of sand–rubber mixtures, particularly in terms of the damping ratio. A series of cyclic triaxial tests were, therefore, performed under a large range of shear strains on sand–rubber mixtures with varying rubber volume contents, rubber particle sizes, and confining pressures. The results indicate the dynamic shear modulus decreases with increasing rubber volume content and with decreasing particle size and confining pressure. The relationship of the damping ratio to the evaluated parameters is complicated and strain-dependent; at shear strains less than a critical value, the damping ratio increases with increasing rubber volume content, whereas the opposite trend is observed at greater shear strains. Furthermore, sand–rubber mixtures with different rubber particle sizes exceed the damping ratio of pure sand at different rubber volume contents. A new empirical model to predict the maximum shear moduli of mixtures with various rubber volume contents, rubber particle sizes, and confining pressures is accordingly proposed. This study provides a reference for the design of sand–rubber mixtures in engineering applications.

## 1. Introduction

The quantity of used rubber tires is increasing every year. It is reported that in just the United States, approximately 290 million old tires are discarded annually [1], approximately 40% of which are disposed in landfills or stockpiles without being effectively utilized [2,3,4]. This excessive accumulation threatens both environmental and human health [5,6]. However, the increasing volume of discarded rubber tires has garnered interest in developing new methods for reusing these materials [7,8,9], and thus motivated researchers to characterize the general geotechnical properties of sand–rubber mixtures [10,11,12,13,14,15,16,17,18,19,20].

Owing to the excellent engineering characteristics of rubber/shredded tires (low volume density, high elastic deformation, and high damping capacity), several previous studies have proposed using such material for earthquake mitigation purposes by, for example, providing damping to foundations or increasing the liquefaction resistance of backfill [21,22,23,24,25,26,27]. However, laboratory experiments thus far conducted to investigate the dynamic behavior of sand–rubber mixtures have not been comprehensive. In particular, only a few previous studies have investigated behaviors of rubber-containing sands under a large range of shear strain amplitudes using cyclic triaxial tests, instead typically focusing on applying small strain levels using resonant column or bender element devices.

Various studies on the dynamic behavior of sand–rubber mixtures have been conducted in recent decades. They can be summarized as follows: Pistolas et al. [28] clearly showed that shear strain has a significant influence on the damping ratio. Sarajpoor et al. [7] reported that at shear strain amplitudes less than about 0.1%, an increase in rubber volume content results in a higher damping ratio, while at shear strain amplitudes greater than about 0.1%, the opposite trend is observed. While Senetakis et al. [21] showed similar conclusions, it should be noted that Okur et al. [29] obtained contrary findings using resonant column tests, indicating that under shear strain amplitudes less than about 5 × 10^−2^%, an increase in rubber volume content leads to a lower damping ratio, with the reverse being true under greater shear strains. Madhusudhan et al. [30] even reported that the damping ratios of sand–rubber mixtures decrease with increasing shear strain range. Thus, although some studies have been conducted to investigate the dynamic behavior of sand–rubber mixtures and have obtained preliminary results, there remain some ambiguous and even contradictory results, particularly for the damping ratio. However, empirical models have been successfully applied to rapidly evaluate other dynamic characteristics of sand–rubber mixtures in engineering applications. For example, Nakhaei et al. [31] introduced a function to predict the maximum shear modulus for various confining pressures and granulated rubber percentages, but concentrated on low rubber volume contents (i.e., 8%, 10% and 14%).

In addition to the rubber volume content, confining pressure and particle size have significant effects on the dynamic behavior of sand–rubber mixtures. For example, Youwai et al. [32] reported that when the particle size ratio *D_rubber_*/*D_sand_* ≤ 6, particle size effects should not be ignored. Lopera Perez et al. [33] performed a series of numerical discrete element method simulations and found that different size ratios have positive or negative effects on the strength and deformability.

In the light of the above discussion, a series of cyclic triaxial tests were performed in this study using a Global Digital Systems (GDS) dynamic cyclic triaxial apparatus in order to improve understanding of the dynamic behavior of sand–rubber mixtures subjected to large shear strain ranges and to clarify previously contradictory observations. Accordingly, the effects of different parameters including the rubber volume content, confining pressure, and rubber particle size on the shear modulus and damping ratio of different sand–rubber mixtures were investigated in detail under large shear strain amplitudes. Furthermore, a new empirical model was proposed and confirmed to predict the maximum dynamic shear moduli of sand–rubber mixtures subjected to various confining pressures (i.e., 50 kPa, 100 kPa, 150 kPa and 200 kPa) with various rubber volume contents (i.e., 10%, 20%, 30%, 40%, and 50%) and different rubber-to-sand particle size ratios.

## 2. Materials and Experimental Methods

### 2.1. Raw Materials

**Figure 1:**
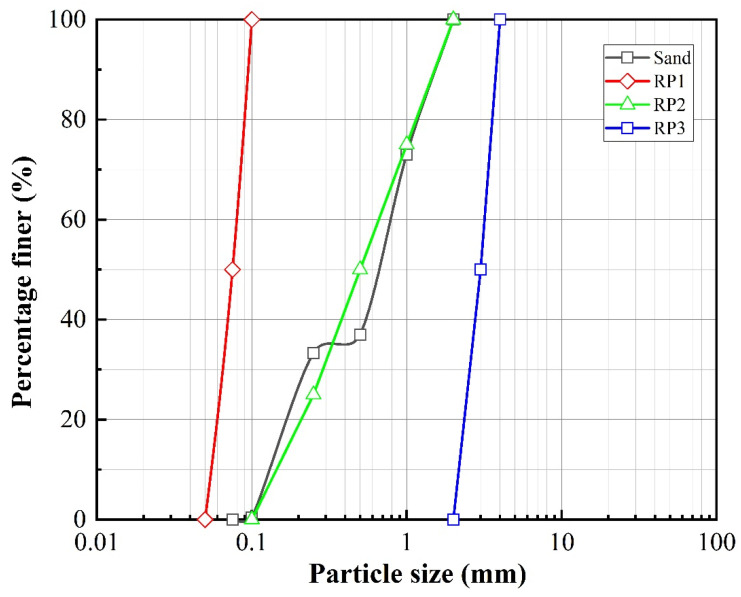
The particle size distribution curves of the tested materials.

Xiamen standard sand with grain sizes from 0.075 mm to 2 mm was used as the host soil to prepare the sand–rubber mixtures in these tests. The waste rubber particles (RP) were provided by a local company specializing in the decomposition of waste rubber tires. After screening, rubber particles sized from 0.05 mm to 4 mm were selected and classified as rubber particles according to ASTM D6270-17 [34]. In order to study the influence of rubber grain size on the dynamic response of the sand–rubber mixtures, the rubber particles were divided into three different grain size ranges: RP1, with a particle size distribution of 0.05–0.1 mm, RP2, with a particle size distribution of 0.1–2 mm, and RP3, with a particle size distribution of 2–4 mm. The particle size distribution curves of the sand and rubber particles are shown in Figure 1, and their main physical properties are provided in Table 1.

In Table 1, *SR* (size ratio) stands for the mean rubber-to-sand particle size ratio and can be determined as follows:

(1) $$SR=\frac{{\left(D_{50}\right)}_{rubber}}{{\left(D_{50}\right)}_{sand}}$$

The *SR* value was used to quantify the rubber particle size ratios for RP1, RP2, and RP3, indicating mean rubber particle sizes respectively larger than, approximately consistent with, and smaller than the mean sand particle size.

### 2.2. Sample Preparation and Mixture Designs

Before preparing the sample, the standard sand was placed in an oven to dry at 100 °C and the rubber particles were air-dried at room temperature (20 ± 2 °C) for 12 h [35]. Afterward, the sand and rubber particles were uniformly mixed for five minutes according to the target rubber volume percentage (*RV*) at room temperature (20 ± 2 °C). The *RV* values considered in this study were 0%, 10%, 20%, 30%, 40%, and 50% determined using the following Equation (2):

(2) $$RV=\frac{V_{r}}{V_{r}+V_{s}}\times 100\%$$

where *V_r_* is the volume of rubber particles and *V_s_* is the volume of sand in the mixture.

The relative density of the specimens, *D_r_*, was determined by:

(3) $$D_{r}=\frac{\rho_{d\max}\times \left(\rho_{d}-\rho_{d}{}_{\min}\right)}{\rho_{d}\times \left(\rho_{d}{}_{\max}-\rho_{d}{}_{\min}\right)},$$

where *ρ_dmax_* and *ρ_dmin_* are respectively the maximum and minimum dry densities of the specimen and *ρ_d_* is its controlled dry density. The maximum dry density and minimum dry density of the different mixtures were respectively obtained by vibratory hammering and the funnel measuring cylinder method according to ASTM D4254 [36].

In this study, a series of 38 mm diameter, 76 mm high specimens were prepared using dry tamping. According to the dry weight of each specimen and the volume of the mold, each mixture was divided evenly into four parts and sequentially compacted in four layers from the bottom to the top of the latex film of the mold. Each specimen was then compacted by tapping the cylinder wall and then vibrating it for two minutes. The relative density was consistently maintained at 0.5. After the specimens were cast, their surfaces were leveled and capped with filter paper and permeable stone. After loading the specimen at the base of the confining chamber, negative pressure was applied to keep it upright.

### 2.3. Test Equipment and Process

A GDS cyclic triaxial testing apparatus was utilized in this study to characterize the dynamic behavior of the sand–rubber mixture specimens, which were manufactured by the Global Digital Systems Ltd. Instrument, Hampshire, United Kingdom. The maximum axial load, operating frequency and pressure of the apparatus are 10 kN, 5 Hz and 2 MPa respectively. The cyclic triaxial apparatus was able to saturate and consolidate the sample under the expected lateral and axial pressures. Then, cyclic axial loading was applied at the top of the specimen, inducing periodic changes in the specimen shear stress.

First, a minimal confining pressure was applied to the specimen and CO_2_ gas was passed through it for at least 30 min to facilitate the saturation process. Then, the specimen was saturated with de-aired water and back pressure until the pore pressure coefficient (*B*-value) increased to greater than 0.95. Next, isotropic pressure consolidation was conducted according to the target mean effective confining pressures (50 kPa, 100 kPa, 150 kPa, and 200 kPa) by controlling the radial stress applied to the inner and outer side walls of the specimen as well as the applied axial stress. It should be noted that if the volume of the back pressure in the specimen remained stable for 5 min after closing the drainage valve, the consolidation was considered to have been completed. Following the consolidation process, the specimen was subjected to stress-controlled cyclic triaxial testing under undrained conditions using a sine wave loading pattern that increased stepwise in amplitude to determine its dynamic parameters. As per ASTM D3999 [37], the cyclic testing was carried out under various axial stress at a loading frequency of 1 Hz until the axial strain exceeded a maximum closure error of 0.2%.

**Figure 2:**
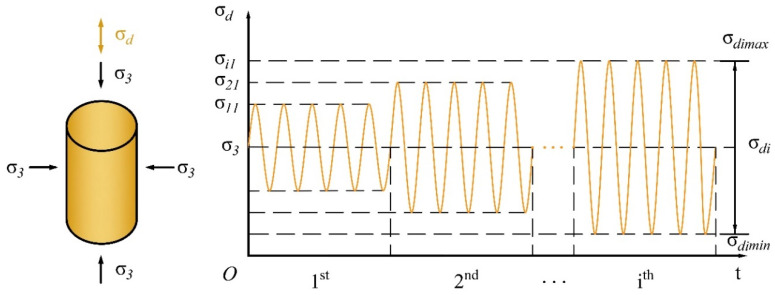
Application diagram of multistage cyclic axial loading.

**Figure 3:**
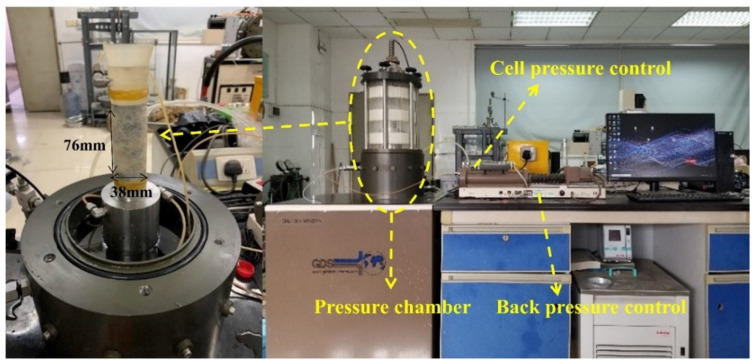
Typical sample diagram and experimental apparatus.

As shown in the multistage cyclic axial loading diagram in Figure 2, all specimens were subjected to five loading cycles in each cyclic loading level. A total of 50 data points were collected for each sinusoidal cycle. The experimental program employed in the current study is summarized in Table 2. Figure 3 shows photographs of the sample diagram and experimental apparatus. It should be noted that according to ASTM D3999 [37], after the completion of each loading step and before moving on to the next higher cyclic load, the specimen drainage valves were opened to re-establish the effective consolidation stress before re-closing them to again impose undrained conditions.

## 3. Constitutive Relation and Parameters

The axial stress and strain in a specimen can be obtained from its response to increasing dynamic loading. It is noted that the true stresses are shown in this study depending on the GDS cyclic triaxial testing apparatus. The maximum value of the results in the third loading cycle of each loading level were, accordingly, used to determine the maximum dynamic shear stress *τ_d_* and dynamic strain *γ_d_* in the specimens according to the following Equations (4) and (5):

(4) $$\tau_{d}=\frac{\sigma_{d}}{2}$$

(5) $$\gamma_{d}=\left(1+\upsilon \right)\times \varepsilon_{d}$$

where *σ_d_* is the maximum dynamic stress in the specimen; *ε_d_* is the maximum dynamic strain in the specimen; and *ν* is the Poisson’s ratio (assumed to be equal to 0.5 in this study [38]).

**Figure 4:**
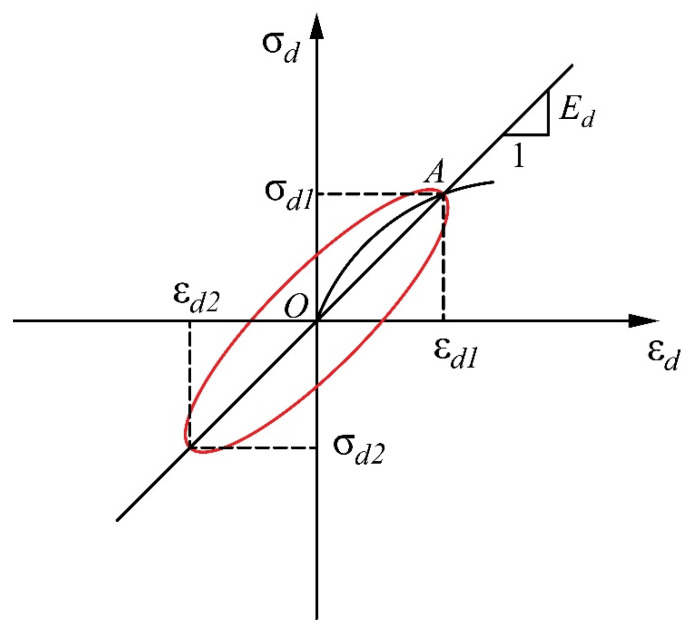
Stress–strain behavior of soil under cyclic axial loading and determination of dynamic elasticity modulus.

Figure 4 presents the stress–strain behavior of a soil under cyclic axial loading and illustrates the determination of the dynamic elasticity modulus. In this study, the dynamic elasticity modulus was calculated as follows:

(6) $$E_{d}=\frac{\sigma_{d}}{\varepsilon_{d}{}_{}}=\frac{\left(\sigma_{d1}-\sigma_{d2}\right)/2}{\left(\varepsilon_{d}{}_{1}-\varepsilon_{d}{}_{2}\right)/2}$$

where *σ_d_*_1_, *σ_d_*_2_, *ε_d_*_1_, and *ε_d_*_2_ are maximum values of axial compressive stress, axial tensile stress, axial compressive strain, and axial tensile strain, respectively. Therefore, the dynamic shear modulus of a specimen is defined as:

(7) $$G_{d}=\frac{E_{d}}{2\times \left(1+\upsilon \right)}$$

**Figure 5:**
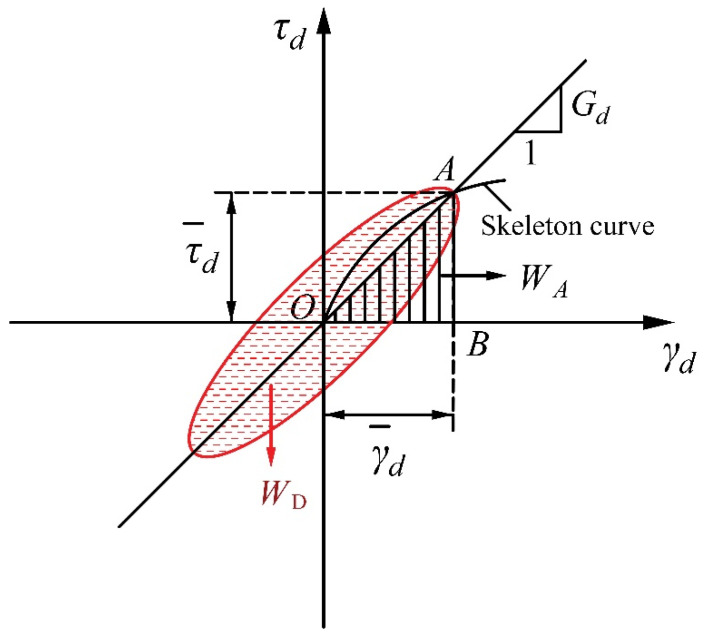
Determination of dynamic shear modulus and damping ratio of a sand–rubber mixture.

The damping ratio is an important dynamic parameter of a soil that expresses the hysteresis characteristics of its stress–strain behavior under cyclic loading. It also reflects the dissipation of energy. As can be seen from the Figure 5, the damping ratio λ can be defined with Equation (8) according to Hardin and Drmevich [39].

(8) $$\lambda =\frac{W_{D}}{4\pi W_{A}},$$

where *W_D_* is the area within the hysteresis loop and *W_A_* is the area of the triangle expressing each cycle.

Hardin and Drnevich [39,40] used the hyperbolic model to describe the relationship between the cyclic shear stress and cyclic shear strain (i.e., skeleton curves). The Hardin model is expressed as follows:

(9) $$\tau_{d}=\frac{\gamma_{d}}{a+b\gamma_{d}},$$

where *ε_d_* is the dynamic shear stress in the sample; *γ_d_* is the dynamic shear strain in the sample; and *a b* are fitting parameters, where *a >* 0 and *b* > 0. The dynamic shear modulus can then be obtained using the dynamic shear stress and strain by:

(10) $$G_{d}=\frac{\tau_{d}}{\gamma_{d}}=\frac{1}{a+b\gamma_{d}}$$

Then:

(11) $$G_{d\max}|{}_{\gamma_{d}=0}=\frac{1}{a}$$

(12) $$\tau_{dult}|{}_{\gamma_{d}=+\infty }=\frac{1}{b}$$

Considering Equations (11) and (12), the model proposed in this study to predict the *γ_dr_* values is given by:

(13) $$\gamma_{dr}=\frac{\tau_{dult}}{G_{d\max}}=\frac{a}{b}$$

where *G_dmax_* is the maximum dynamic shear modulus, *τ_dult_* is the ultimate dynamic shear stress magnitude, and *γ_dr_* is the reference shear strain magnitude. Consequently, the 1/*G_d_* vs. *γ_d_* curve can be plotted from the experimental data to obtain and the fitting parameters *a* and *b*. Next, by substituting Equations (11)–(13) into Equation (10):

(14) $$G_{d}=\frac{G_{d\max}}{1+{\gamma_{d}/\gamma }_{dr}}$$

(15) $$\frac{G_{d}}{G_{d\max}}=\frac{1}{1+{\gamma_{d}/\gamma }_{dr}}$$

Thus, it can be determined from Equations (14) and (15) that the dynamic shear modulus of a sand–rubber mixture is a function of the dynamic shear strain.

## 4. Dynamic Cyclic Triaxial Test Results

In this study, the dynamic behavior of sand–rubber mixture specimens, including their dynamic shear stress–strain relationships, shear moduli, and damping ratios under different strain ranges, were determined by applying an increasing shear stress amplitude. These dynamic parameters were obtained for specimens with various rubber volume contents, rubber particle sizes, and confining pressures.

### 4.1. Dynamic Shear Stress–Strain Relationship

**Figure 6:**
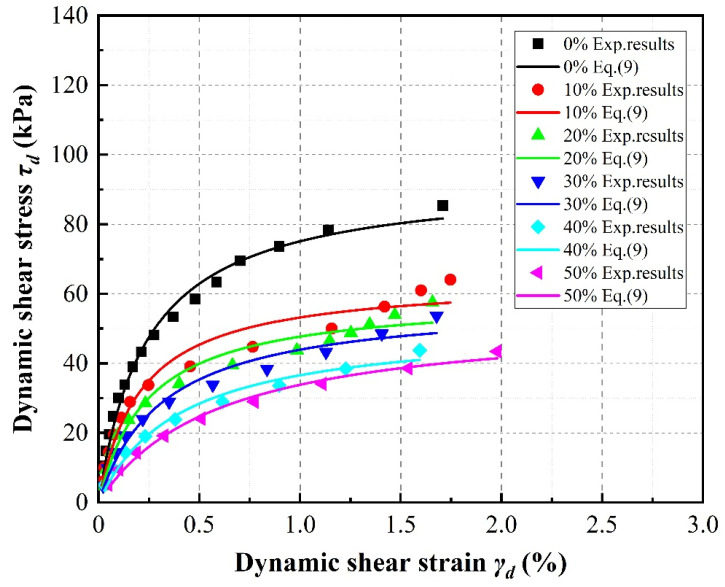
Effect of rubber content on stress–strain relationships of SRP2 sand–rubber mixtures under 100 kPa confining pressure.

**Figure 7:**
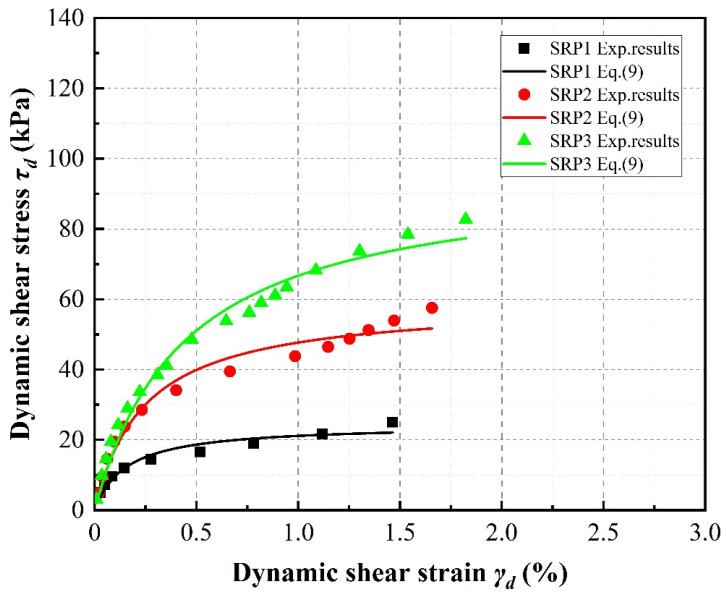
Effect of rubber particle size on stress–strain relationships of sand–rubber mixtures with 20% rubber content under 100 kPa confining pressure.

**Figure 8:**
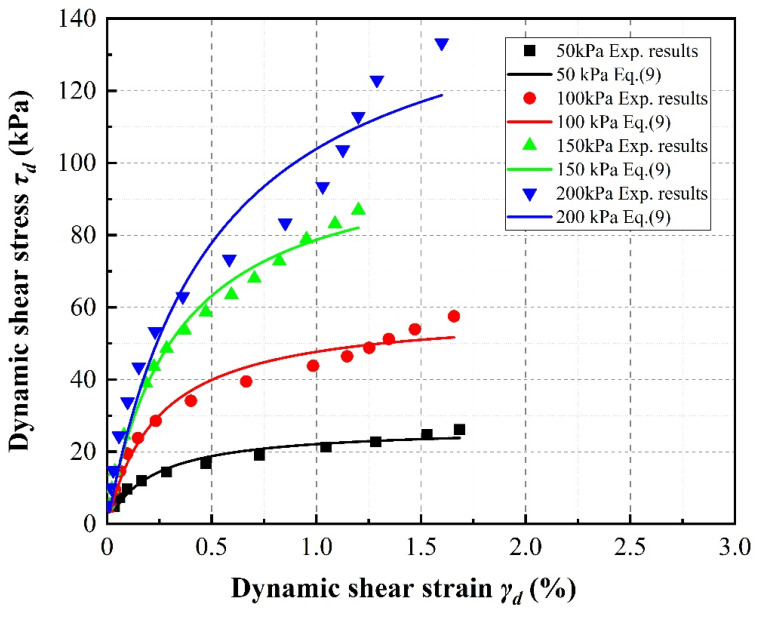
Effect of confining pressure on stress–strain relationships of SRP2 sand–rubber mixtures with 20% rubber content.

Figure 6, Figure 7 and Figure 8 present the dynamic diaphysis curve, which can be obtained by the connecting peaks of the shear stress–strain hysteresis loops for different loading steps. Generally, all specimens exhibit strain-hardening behavior under these stress-controlled consolidated undrained triaxial tests; that is, the maximum dynamic shear stress is observed at a large strain. The general trend observed in these figures appears to follow the well-known hyperbolic law. It can also be observed that, at shear strain amplitudes less than about 0.5%, the dynamic shear stress increases and the dynamic shear strain is nearly constant, exhibiting only a slight increase, whereas the shear strain increases obviously at shear strain amplitudes higher than about 0.5%.

In the case of SRP2 under a confining pressure of 100 kPa, it can be observed in Figure 6 that the dynamic diaphysis curves gradually shift downward with increasing rubber volume content, indicating that under a constant shear strain, the specimen with the higher rubber volume content exhibits a lower shear stress. In addition, note that the downward shift in the dynamic shear stress curves gradually decreases between each evaluated rubber volume content. The dynamic diaphysis curve first shifts rapidly downward due to an increase in rubber volume content from 0% to 10%, but this change slows or even disappears for increases between 20% and 50%, which is similar to the results of Madhusudhan et al. [30] and Mashiri et al. [41].

Figure 7 indicates that the rubber particle size also affects the dynamic diaphysis curve. Taking the case of 20% rubber volume content under a confining pressure of 100 kPa as an example, an increase in particle size leads to an upward shift in the dynamic diaphysis curve. Thus, the shear stress increases dramatically with increasing rubber particle size under a confining pressure of 100 kPa.

As illustrated in Figure 8, taking the case of 20% SRP2 rubber volume content as an example, an increase in confining pressure also leads to an upward shift in the dynamic diaphysis curve. It can be noted that this phenomenon is clearer under low confining pressures (i.e., from 50 to 100 kPa) than under high confining pressures (i.e., from 150 to 200 kPa).

### 4.2. Dynamic Shear Modulus

**Figure 9:**
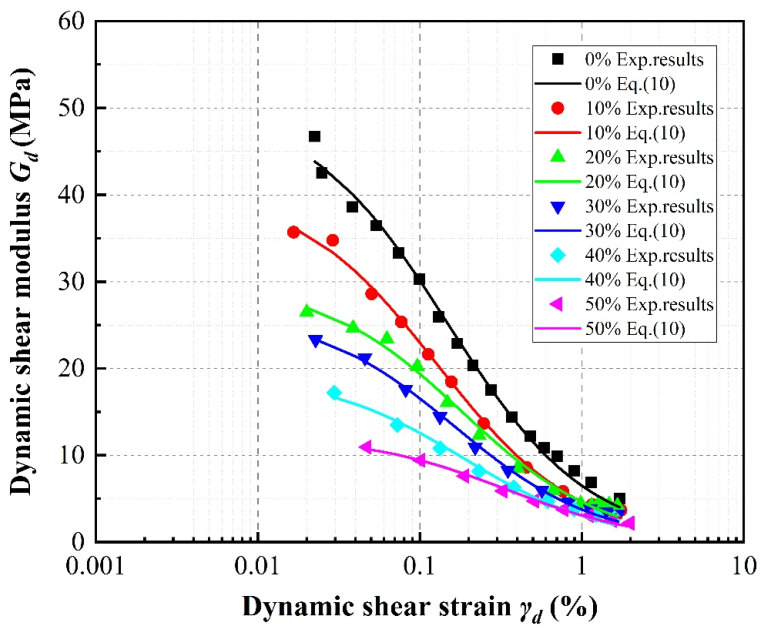
Effect of rubber content on dynamic shear moduli of SRP2 sand–rubber mixtures under 100 kPa confining pressure.

**Figure 10:**
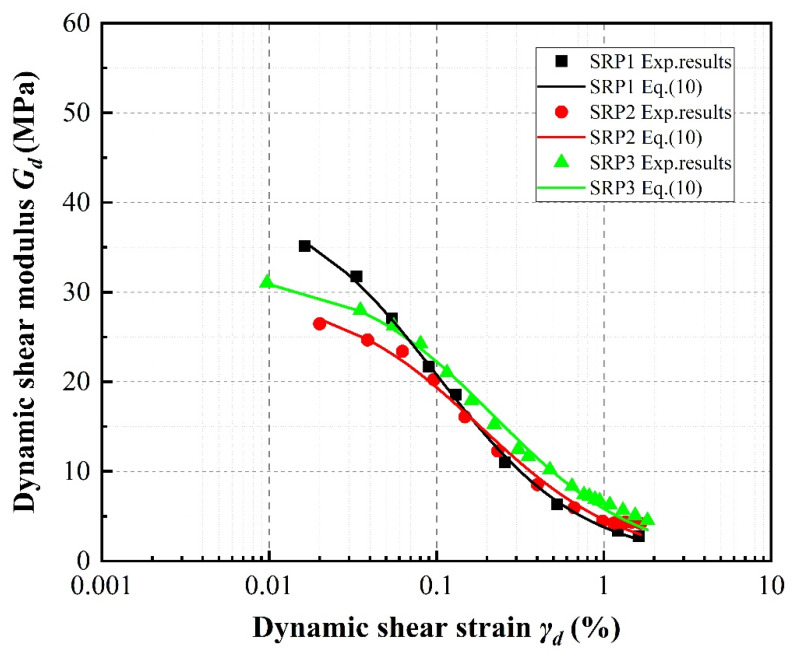
Effect of rubber particle size on dynamic shear moduli of sand–rubber mixtures with 20% rubber content under 100 kPa confining pressure.

**Figure 11:**
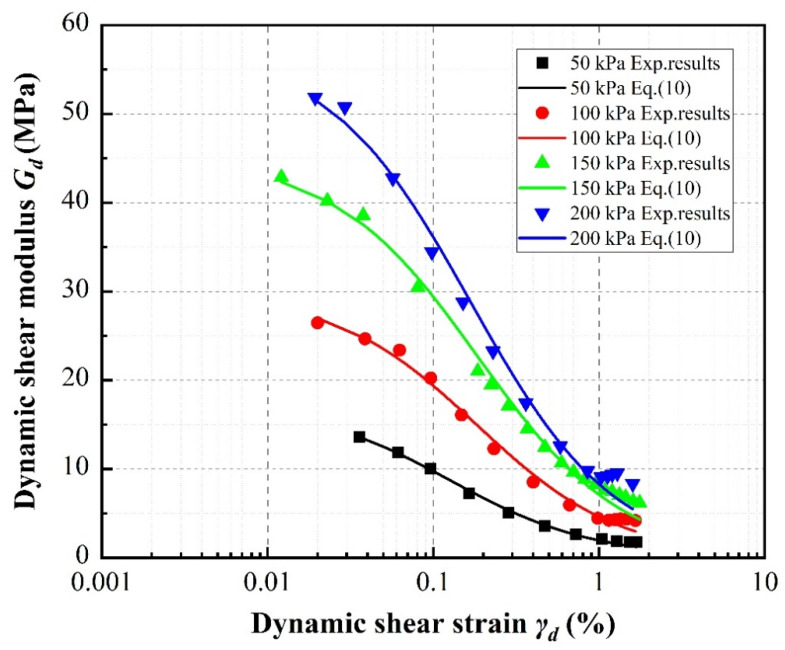
Effect of confining pressure on dynamic shear moduli of SRP2 sand–rubber mixtures with 20% rubber contents.

Figure 9, Figure 10 and Figure 11 compare the experimental data obtained for the dynamic shear modulus under the different evaluated conditions. In general, the dynamic shear modulus significantly decreases with increasing shear strain amplitudes less than 0.5%, then slows at shear strains greater than 0.5% before settling at a low value. It is noted that the specimens containing rubber particles exhibit lower dynamic shear modulus values than pure sand. Moreover, the distribution of dynamic shear modulus data is concentrated at larger strains regardless of rubber volume content, with the dynamic shear modulus values of the specimens with different rubber contents even partially overlapping at the highest strains. This behavior is similar to that of the stress–strain relationship described above.

It can be observed in Figure 9, which takes SRP2 under a confining pressure of 100 kPa as an example, that as the rubber volume content increases, the initial dynamic shear moduli of the specimens at small strain gradually decrease. Obviously, specimens with a lower rubber volume content show higher initial dynamic shear moduli than specimens with higher rubber volume content during dynamic loading. It can further be observed that pure sand exhibits the highest dynamic shear modulus of approximately 46.72 MPa, the dynamic shear modulus of the specimen with 10% rubber volume content is approximately 35.71 MPa (a 23.57% reduction compared to sand), and the dynamic shear modulus of the specimen with 50% rubber volume content is the smallest at approximately 10.93 MPa (a reduction of 76.61% compared to sand). Additionally, note that the dynamic shear moduli of the mixture specimens decrease slightly at higher rubber volume contents. Feng et al. [34] and Sarajpoor et al. [7] have reported similar observations.

Taking the 20% rubber volume content specimen under a confining pressure of 100 kPa as an example, Figure 10 clearly shows that the dynamic shear modulus of each specimen generally increases with increasing rubber particle size. However, the dynamic shear modulus slightly decreases with increasing particle size between SRP2 and SRP1 at strains greater than 0.2%.

Figure 11 demonstrates that as the confining pressure increases, the dynamic shear moduli of the specimens increase. Moreover, the higher the confining pressure, the greater the initial increase in the initial dynamic shear modulus. For SRP2 with 20% rubber volume content, compared to a confining pressure of 50 kPa, the initial dynamic shear modulus increases 94.48%, 214.92%, and 280.09% under confining pressures of 100 kPa, 150 kPa, and 200 kPa, respectively.

### 4.3. Damping Ratio

**Figure 12:**
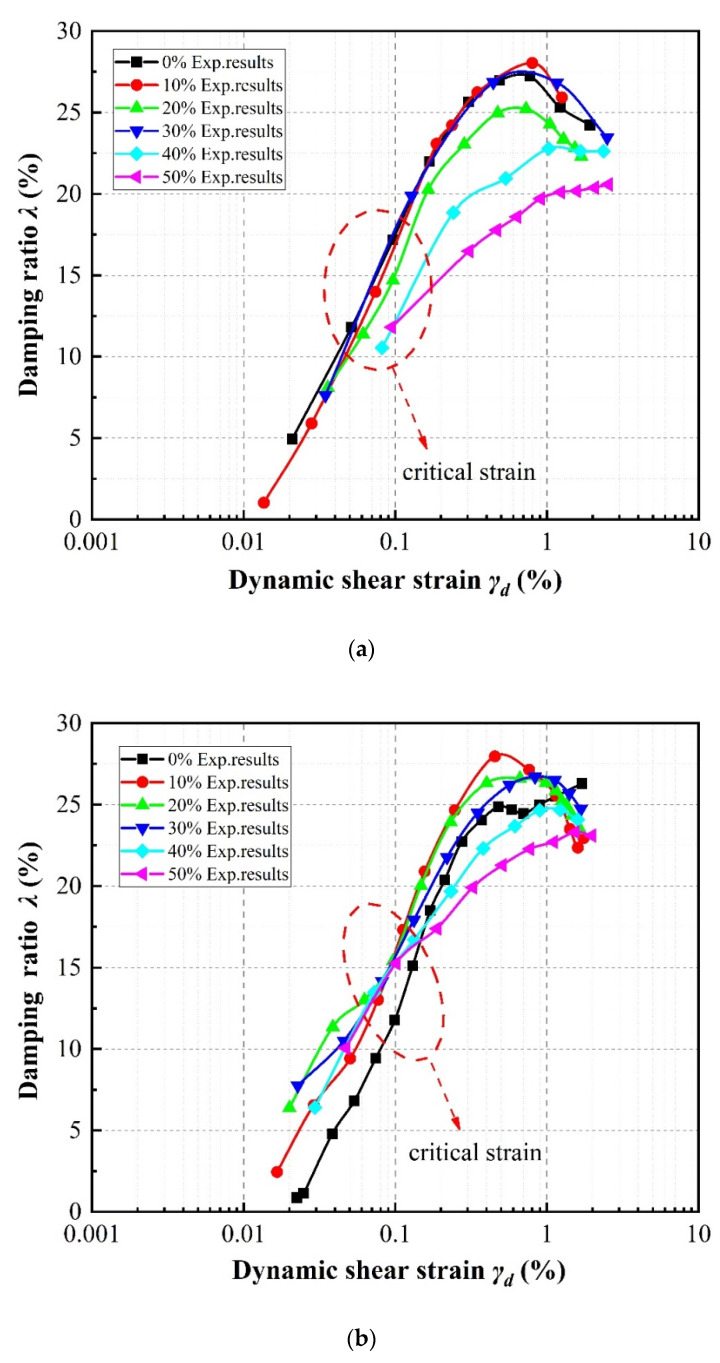

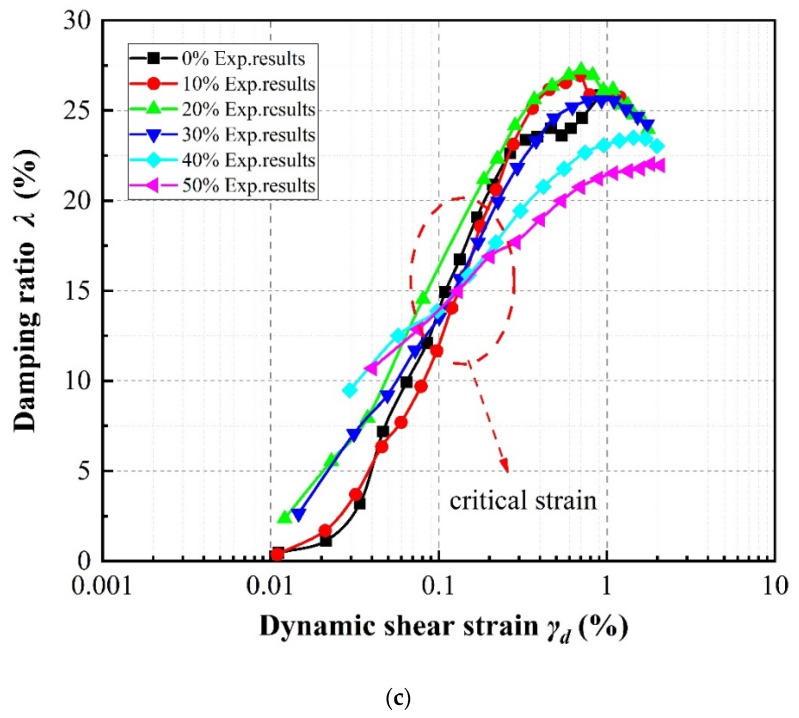
Effect of rubber content on damping ratios of SRP2 sand–rubber mixtures under confining pressures of: (**a**) 50 kPa; (**b**) 100 kPa; (**c**) 150 kPa.

**Figure 13:**
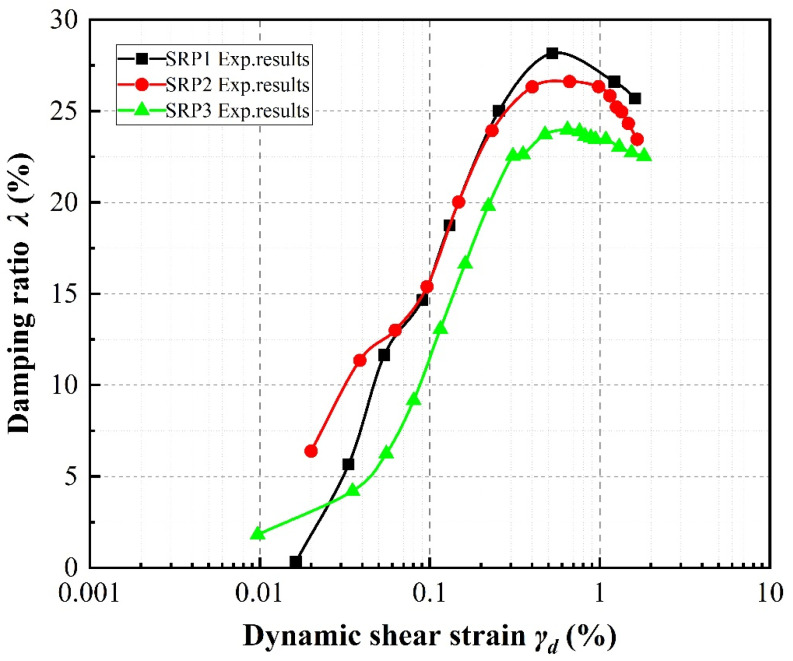
Effect of particle size on damping ratios of sand–rubber mixtures with 20% rubber content under 100 kPa confining pressure.

**Figure 14:**
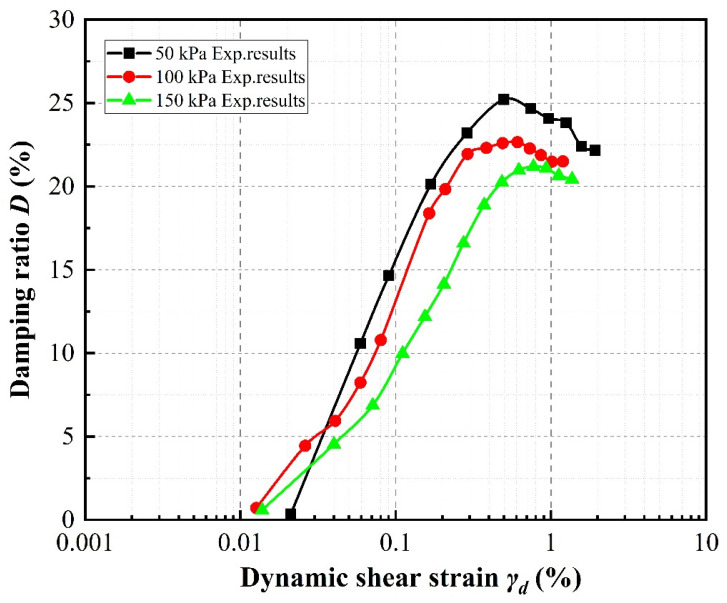
Effect of confining pressure on damping ratios of SRP2 sand–rubber mixtures with 20% rubber volume content.

The variations in damping ratio with shear strain according to specimen parameter are illustrated in Figure 12, Figure 13 and Figure 14. Generally speaking, at shear strain amplitudes less than about 1%, the damping ratios of the specimens increase as the shear strain develops, whereas at shear strain amplitudes greater than about 1%, the damping ratios decrease.

Figure 12 indicates that, taking the case of SRP2 as an example, the damping ratio initially increases with increasing rubber volume content. This also can be observed in the results of Ehsani et al. [42], and is similar to the results of Sarajpoor et al. [7] and Senetakis et al. [21] as previously mentioned. Note that the damping ratio exhibits a strong dependence on the rubber particle size, and shows an optimal value at a specific rubber volume content. For SRP2, the optimal rubber volume content at which the mixture damping ratio is higher than that of pure sand is about 20% to 30%, while in the case of SRP1 (not pictured) the optimal rubber volume content is about 10% to 20%, but for SRP3 (also not pictured), the damping ratio is always lower than that of pure sand. Obviously, the effect of rubber volume content on the damping ratio is influenced by the confining pressure and shear strain. As the rubber volume content increases, the relationship between damping ratio and shear strain tends to remain stable, which indicates a low sensitivity of the damping ratio to variations in shear strain amplitude. It can further be observed in Figure 12b that at shear strain amplitudes less than about 0.1%, a higher rubber volume content leads to a higher damping ratio, whereas at shear strain amplitudes greater than 0.1%, the opposite trend can be observed. The tests conducted at different confining pressure levels further revealed that these critical strain values increase with increasing confining pressure: the critical strain increases from 0.09% to 0.1% to 0.11% for confining pressures of 50 kPa, 100 kPa, and 150 kPa, respectively.

It can be seen in Figure 13, taking the 20% rubber volume content specimens under a confining pressure of 100 kPa as an example, that as the rubber particle size increases, the damping ratios of the mixture specimens decrease. In particular, the damping ratio curve for SRP1 moves close to that for SRP2 and even overlaps, while SRP3 curve remains far away from the other two.

Figure 14 shows that, taking a rubber volume content of 10% and SRP3 particle size as an example, the damping ratio decreases with increasing confining pressure. Thus, the effect of confining pressure on the trend of the curve is similar to that of the rubber particle size.

## 5. Discussion of Experimental Results

**Figure 15:**
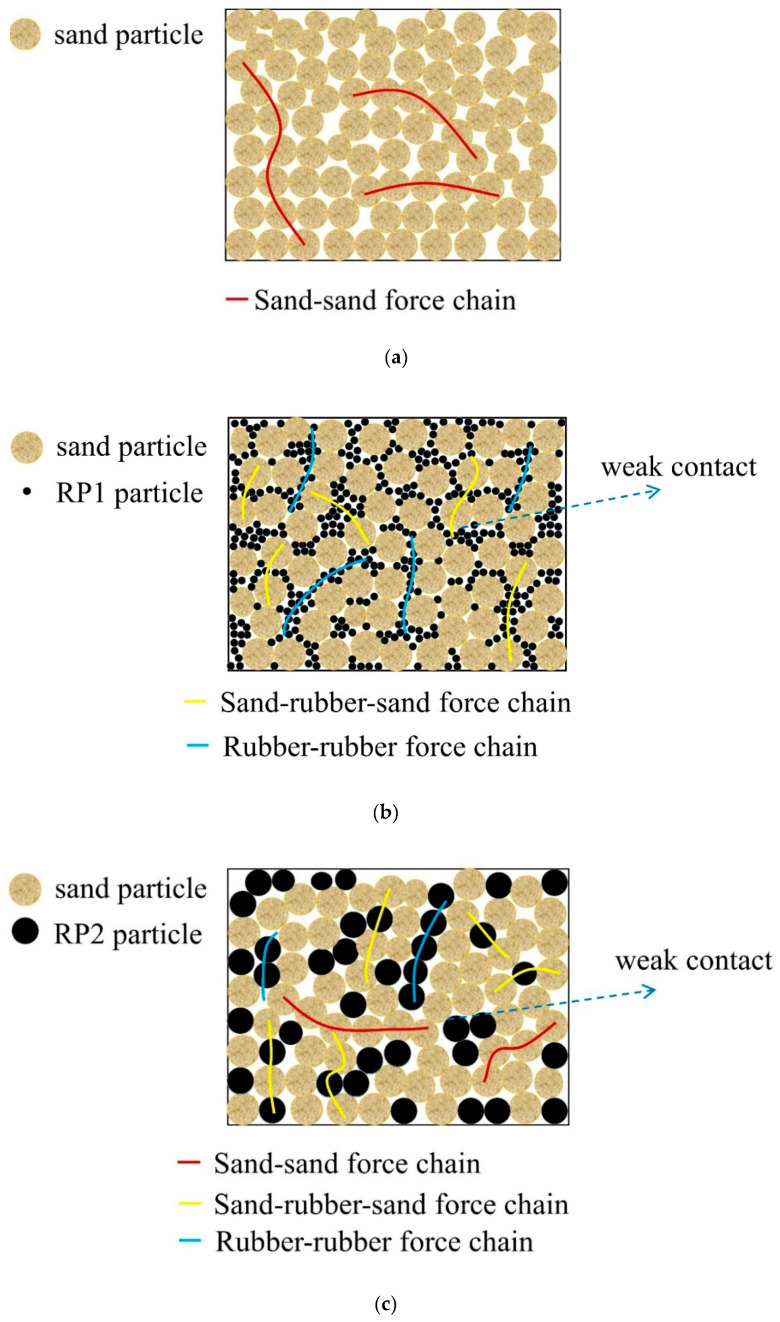

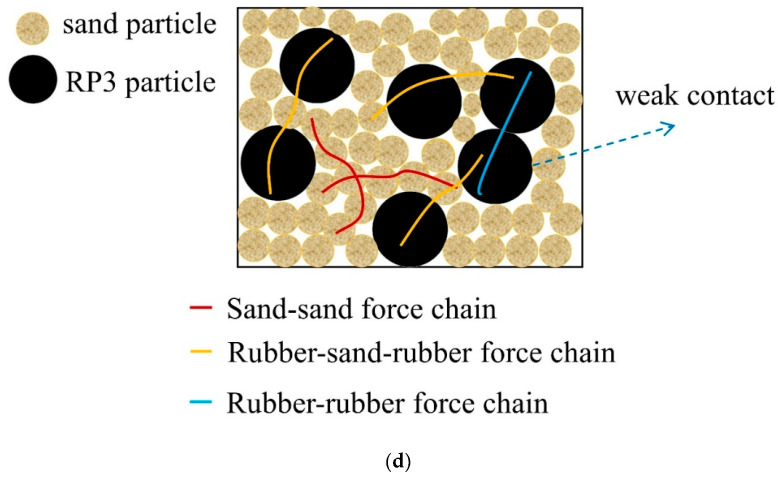
Schematic diagram of internal contact and force transmission chains in (**a**) pure sand; (**b**) SRP1; (**c**) SRP2; (**d**) SRP3.

Rubber particles have low stiffness, strong deformation capacity, and large elasticity, and thus can easily change shape. In contrast, sand particles can be assumed to be rigid particles, especially relative to rubber particles [38,43]. Figure 15 presents schematic diagrams of the internal contact and force transmission chains of the various specimen types evaluated in this study. Note that the structural surface contact weakens due to the addition of rubber particles to sand [44]. As the rubber volume content increases, more rubber particles come into contact with each other, causing the specimen properties to become increasingly rubber-like. This observation is compatible with the results of Liu et al. [11], who proposed different force transmission chains in specimens with different rubber volume contents. Accordingly, the stress–strain properties of a sand–rubber mixture are gradually dominated by the rubber particles as their quantity increases. At a constant rubber volume content and relative density, smaller-sized rubber particles induce an increase in the number of contacts between rubber particles and an decrease in the contact between sand particles, potentially increasing the incidence of sand–rubber–sand and rubber–rubber force transmission chains while decreasing the incidence of sand–sand force transmission chains. This may cause the specimens to exhibit a rubber-like transformation earlier during dynamic loading. Furthermore, it is noted that smaller-sized rubber particles can more easily move relative to one another, thus increasing the plastic strain in the specimen. However, the large inter-particle forces obtained under a high confining pressure inhibit the movement of particles under dynamic loading.

The low stiffness and high elastic deformation of the rubber particles added to the mixtures induce weak contact between specimen particles, influencing the dynamic shear modulus. The incidence of sand–rubber–sand and rubber–rubber force transmission chains increase with increasing rubber volume content and decreasing rubber particle size, potentially increasing the rubber-like behavior of the specimens and reducing the intergranular friction force. Furthermore, the specimens were observed to behave more linearly with increasing rubber volume content, thus their dynamic shear moduli show less sensitivity to the shear strain levels. Lee et al. [45] and Shan et al. [46] reported that the contact between rigid particles increases and particle arrangement is hindered with increasing restraint stress. Hence, an increasing confining pressure considerably improves the restraint stress of a specimen causing it to behave more like pure sand, resulting in a higher shear deformation resistance capacity. These findings can be summarized as follows: when the rubber volume content increases, the rubber particle size decreases, or the confining pressure decreases, the initial dynamic shear modulus of the specimen decreases.

The damping results express the inherent damping provided by the rubber particles, as well as the particle movement and intergranular friction [47]. As the coefficient of friction for rubber is less than that for sand [33], a great deal of energy dissipation results from the intergranular friction between sand particles and their sliding under dynamic loading [47] because pure sand is almost incompressible [48]. In contrast, rubber is a highly elastic material that mainly contributes to the inherent damping of the specimen vibration [22]. However, the damping mechanism is also affected by the shear strain levels. At small strain levels less than the critical strain, the inherent damping provided by the rubber particles contributes more to energy dissipation than other factors, and therefore the sand–rubber mixtures exhibit greater damping than the pure sand. At high strain levels greater than the critical strain, intergranular friction contributes more to energy dissipation than the rubber particles [7]. The effects of rubber volume content, rubber particle size, and confining pressure on damping ratio can be summarized as follows:

1. An increase in the rubber volume content causes some sand particles in the specimens to be replaced by rubber particles, increasing the weak contacts between rubber and sand, in turn decreasing the intergranular friction and leading to a decrease in the damping ratio. At a sufficiently high rubber volume content, the development of more weak contact points is expected to cause the specimens to behave more linearly, leading to a damping ratio that is less sensitive to the variation in the shear strain amplitude. Liu et al. [11] reported that rubber particle begin to consistently contact each other at a medium rubber volume content (20–40%). With increasing incidence of sand–rubber–sand and rubber–rubber force transmission chains, the overall stiffness of the sand–rubber mixture specimens are further decreased and deformability increased due to the elasticity of the more prevalent rubber particles. This explains why the mixtures with rubber volume contents of approximately 20–30% exhibit damping performance superior to that of pure sand in this study.
2. Although the results of Liu et al. [11] illustrate different force transmission chains according to rubber volume content, we determined in this study that the quantities and types of force transmission chains are also influenced by the particle size. As can be seen in Figure 15, when the rubber volume content is kept constant, smaller rubber particles more effectively fill the gaps between sand particles. In other words, the quantity of sand–rubber and rubber–rubber contact points increase, increasing the number of rubber–rubber and sand–rubber–sand force transmission chains, and facilitating the movement of sand particles during shear loading, ultimately leading to higher damping ratio. This finding is compatible with the numerical modelling results obtained by Lopera Perez et al. [33], and demonstrates that rubber volume content and particle size comprehensively affect the damping ratio of a rubber–sand mixture. Accordingly, as the mean rubber particle sizes of SRP2 and SRP1 are smaller than that of pure sand in this study, the damping ratios of these mixture specimens are similar.
3. In pure sand, intergranular friction increases with increasing confining pressure. This mechanism reduces the relative movement between sand particles and subsequently decreases the width of the hysteresis loop, which is a recognized event in geotechnical earthquake engineering literature [31]. However, the flexibility of the added rubber particles facilitates the rearrangement and relative movement of the sand particles, which are influenced by the shear strain level [7]. Hence, there exists a critical shear strain controlling the damping behavior of sand–rubber mixtures that depends on the applied confining pressure. The existence of such a critical strain in this study is compatible with the results of Senetakis et al. [21], which were solely based on experimental data within a shear strain range of 10^−3^% to 5 × 10^−2^%; behavior beyond this range was predicted by curve fitting assuming a hyperbolic model. In this study, tests were conducted over a much wider shear strain range.

It should be noted that because rubber has a lower volume density than sand [33], the distribution of rubber particles will be random and difficult to control in a specimen, which may explain the dispersed nature of the experimentally obtained damping ratio data. Finally, note that the damping ratio values of all specimen types decrease under large strains mainly because the specimen is compacted as the loading proceeds. Overall, the ability to maintain the shear stiffness of the specimen while increasing its damping ratio by adjusting the volume content and particle size of the rubber in the mixture constitutes a prominent advantage of sand–rubber mixtures.

## 6. Empirical Model

An empirical model was developed based on the experimental results to predict the effects of rubber volume content and confining pressure on the behavior of sand–rubber mixtures. In this section, the effects of rubber volume contents, rubber particle size, and confining pressure on the maximum shear modulus are first evaluated based on Equation (11). Then, an empirical model is constructed for the maximum shear modulus as it has a direct influence on the calculation of the reference shear strain and normalized shear modulus. Finally, the empirical model is used to determine the reference shear strain and then predict the normalized shear modulus according to the rubber volume contents, rubber particle size, and confining pressure using Equations (13) and (15).

### 6.1. Maximum Shear Modulus

**Figure 16:**
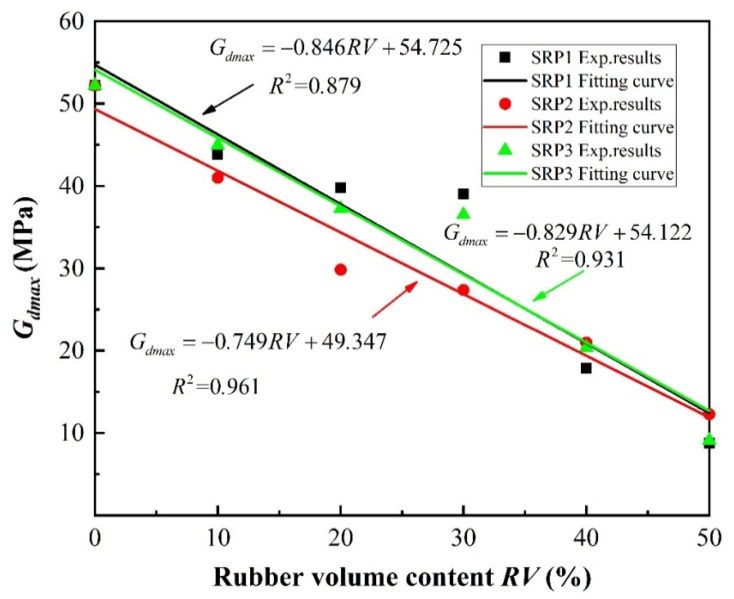
Effect of rubber content on the maximum dynamic shear moduli (*G_dmax_*) of sand–rubber mixture specimens under 100 kPa confining pressure.

**Figure 17:**
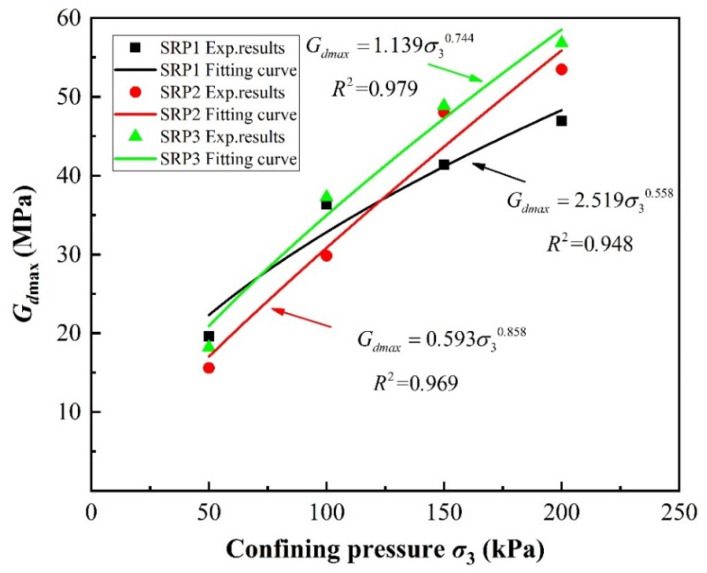
Effect of confining pressure on maximum dynamic shear moduli (*G_dmax_*) of sand–rubber mixtures with 20% rubber volume content.

Figure 16 and Figure 17 show the maximum dynamic shear modulus versus rubber volume content curves under different confining pressures, obtained using Equation (11). It can be seen that the maximum dynamic shear modulus increases as the rubber volume content decreases following an approximately linear relationship. As shown in Figure 16, in the case of SRP1 under a confining pressure of 100 kPa, the maximum dynamic shear moduli of the specimens with 10%, 20%, 30%, 40%, and 50% rubber volume contents were 8.2%, 26.2%, 41.9%, 56.0%, and 67.2%, respectively, less than that of pure sand. This indicates that adding rubber into sand reduces the dynamic shear modulus of the mixture due to the low stiffness of rubber particles, which is consistent with the discussion in Section 5. Thus, the isolation performance of a sand–rubber mixture can be improved by controlling its rubber content. Additionally, the effect of confining pressure on maximum dynamic shear modulus for a given rubber volume content, shown in Figure 17, resembles an exponential function. However, no function could be found using the data collected in this study to describe the effect of rubber particle size on maximum dynamic shear modulus.

### 6.2. Empirical Model and Validation

**Figure 18:**
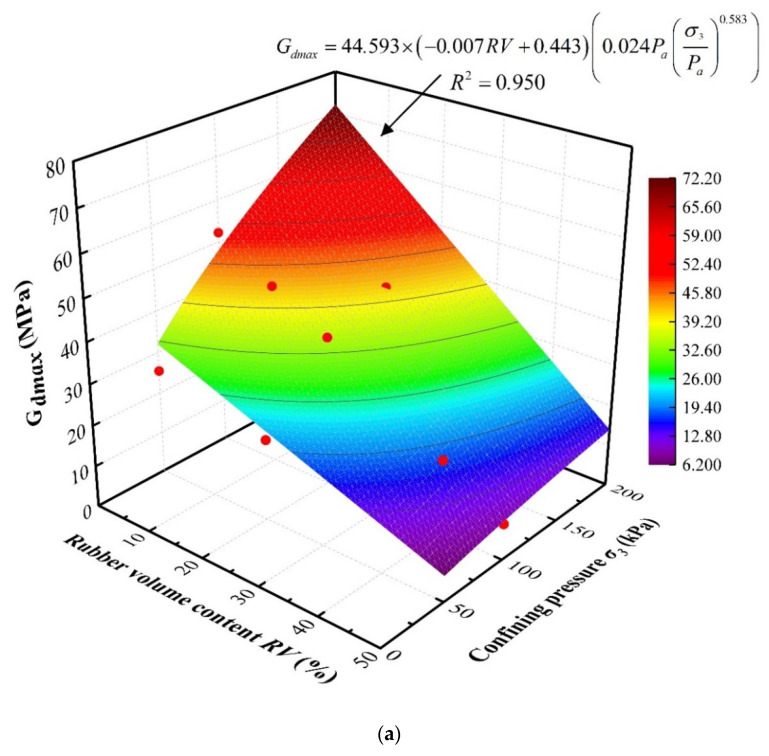

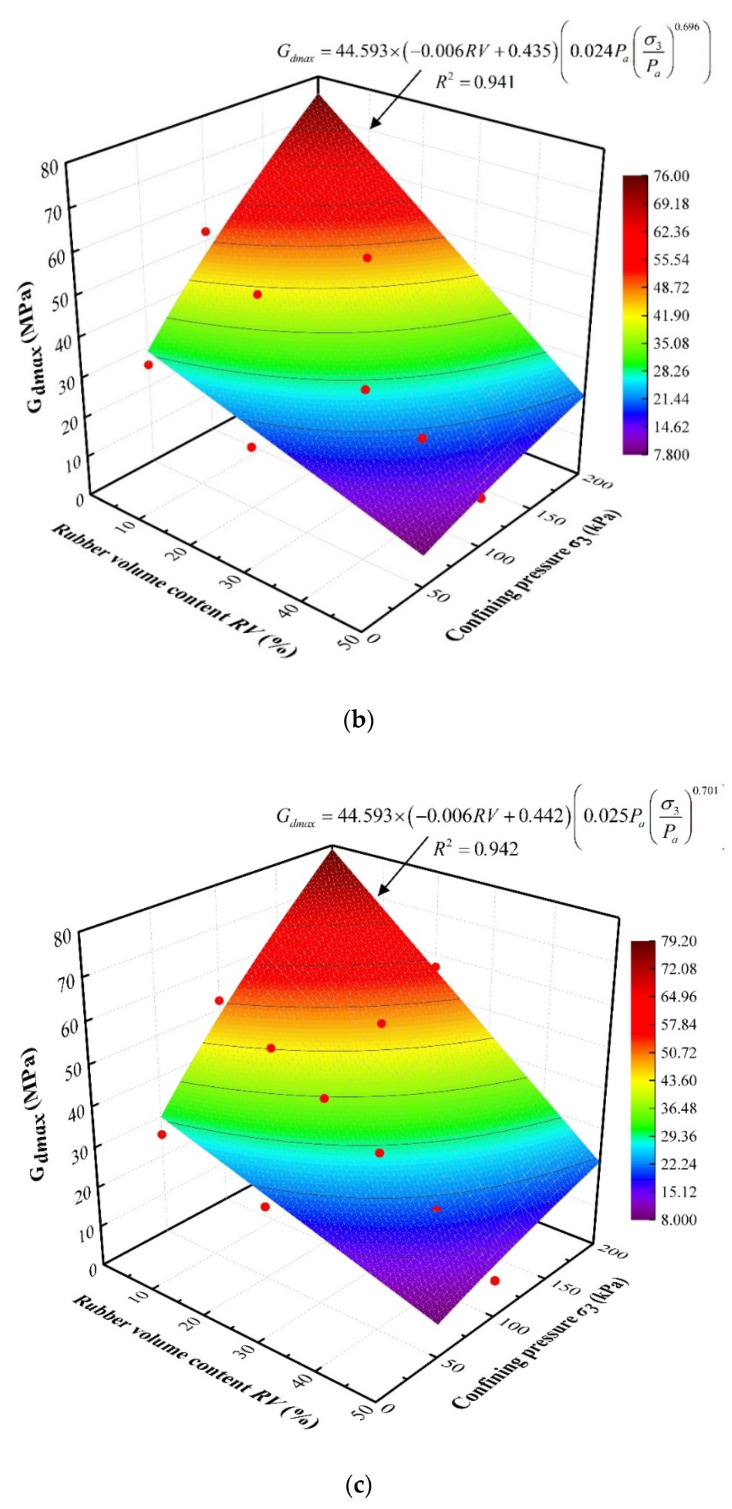
Empirical model of maximum dynamic shear modulus (*G_dmax_*) according to rubber content and confining pressure for different rubber particle sizes: (**a**) SRP1; (**b**) SRP2; (**c**) SRP3.

Equations (13) and (15) indicate that the maximum dynamic shear modulus is an important parameter in the calculation of the reference shear strain and normalized shear modulus, both of which play an important role in the design and evaluation of sand–rubber mixtures. Thus, a comprehensive function describing the maximum shear modulus *G_dmax_* of a sand–rubber mixture according to rubber volume content *RV* and confining pressure *σ*_3_ was obtained by considering the experimental data using the multiple regression analysis method as follows:

(16) $$G_{\mathrm{dmax}}=G_{0}\times \left(\alpha_{i}\times RV+\lambda_{i}\right)\times \left(\theta_{i}\times P_{a}\times {\left(\frac{\sigma_{3}}{P_{a}}\right)}^{\beta_{i}}\right)$$

where *G*_0_ is the model parameter, defined as the averaged maximum shear modulus of pure sand under the three different evaluated confining pressures; *α_i_*, *λ_i_*, *θ_i_*, and *β_i_* are fitting parameters; *i* indicates the rubber particle size, equal to 1, 2, and 3 for SRP1, SRP2, and SRP3, respectively; and *P_a_* is the reference stress taken as equal to the atmospheric pressure, 101.3 kPa. The fitting parameters were determined using a non-linear surface regression analysis of the experimental data obtained by the laboratory experiments presented in Section 3, Section 4 and Section 5, and the empirical function results are, accordingly, plotted in Figure 18.

**Figure 19:**
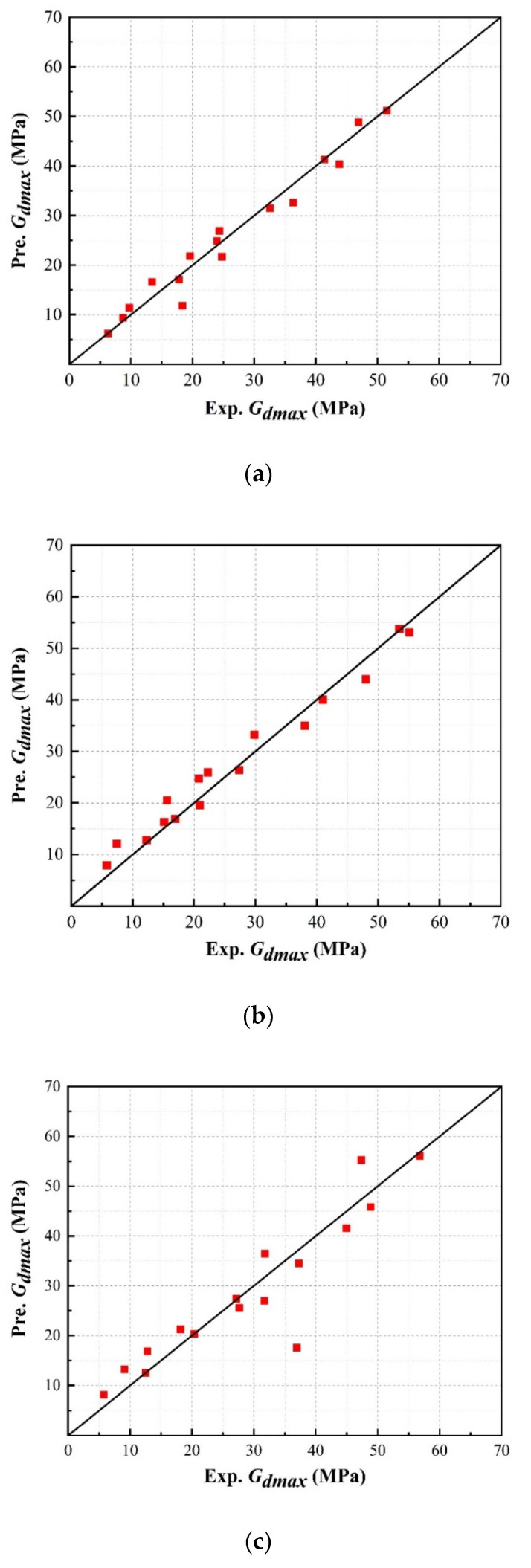
Validation of empirical model results (Pre.) against experimental results (Exp.) for different rubber particle sizes: (**a**) SRP1; (**b**) SRP2; (**c**) SRP3.

Figure 19 compares the maximum dynamic shear modulus predicted using Equation (16) with the experimental data, in which it can be observed that the predicted results for the dynamic shear modulus present a linear correlation of roughly 1:1 with the experimental results. This indicates that Equation (16) can be confidently utilized to estimate and predict the maximum dynamic shear modulus of a sand–rubber mixture.

### 6.3. Reference Shear Strain and Normalized Shear Modulus

The attenuation of the shear modulus is an important dynamic behavior of sand–rubber mixtures. The verification of Equation (16) in Section 6.2 indicates that the proposed empirical model can be confidently utilized to predict the maximum dynamic shear modulus of a sand–rubber mixture, but in order to clearly illustrate the effects of rubber volume content, rubber particle size, and confining pressure on the attenuation of the shear modulus, it is necessary to first determine the reference shear strain and then normalized shear modulus. In this section, the proposed empirical model is, therefore, used to determine the reference shear strain and normalized shear modulus using its predicted value for maximum shear modulus in Equations (13) and (15), respectively.

**Figure 20:**
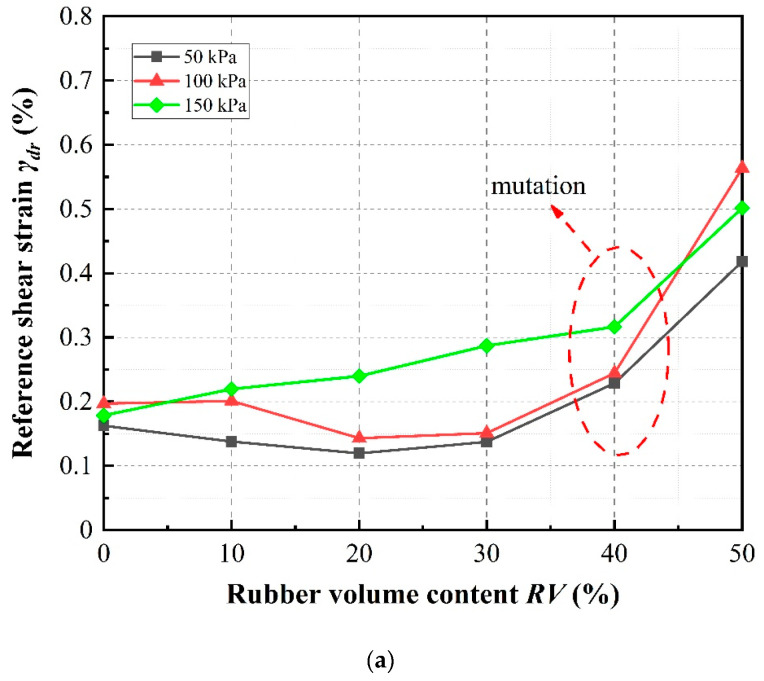

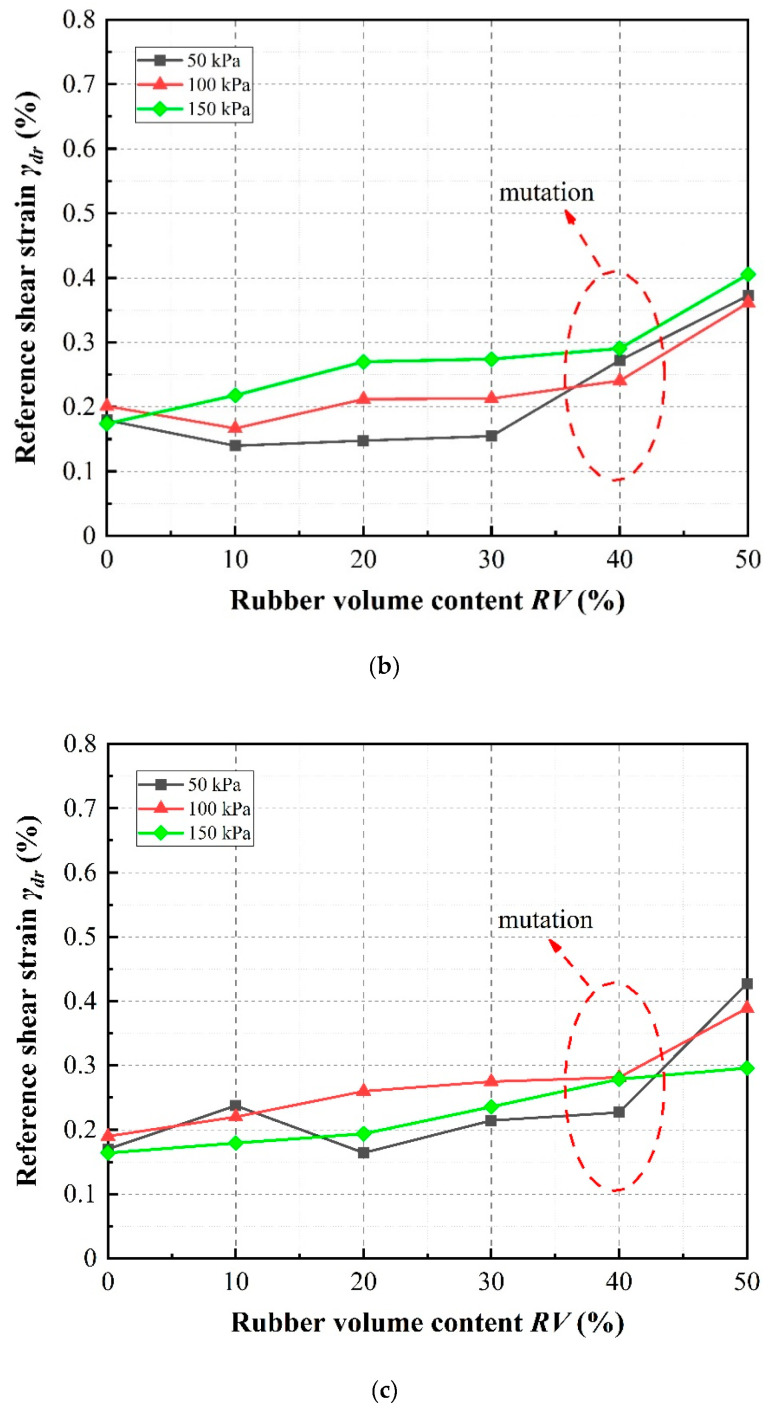
Effect of rubber volume content and confining pressure on reference shear strain of sand–rubber mixtures with different rubber particle sizes: (**a**) SRP1; (**b**) SRP2; (**c**) SRP3.

Figure 20 shows that the reference shear strain obtained using Equations (13) and (16) almost increases with increasing rubber volume content under different confining pressures. The reference shear strain can be observed to increase significantly under a confining pressure of 150 kPa, while several opposite trends can be observed under confining pressure of 50 kPa and 100 kPa especially for low rubber content. Furthermore, it is also observed that, at rubber volume contents less than about 40%, the reference shear strain, almost exhibiting only a slight increase with increasing rubber volume content, whereas the shear strain increases considerably with increasing rubber volume content values greater than 40%. These observations can be explained by the fact that more rubber particles come into contact with each other with increasing rubber volume content and confining pressure. The stress–strain relationship of the mixtures becomes more similar to that of pure rubber, indicating the development of rubber-like behavior. This observation is compatible with the results of Liu et al. [11]. Therefore, the slope of the stress–strain diagram decreases, resulting in an increase in the reference shear strain values. While under low rubber volume content and confining pressure, these contacts were not obvious, the reference shear strain values increase inconspicuously and even appear to be on a converse trend.

**Figure 21:**
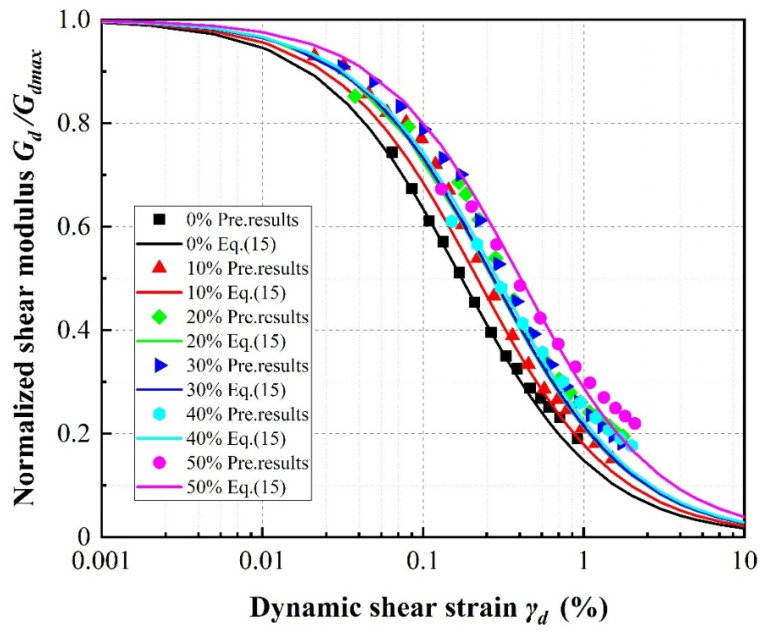
Normalized shear modulus versus shear strain for SRP2 under 150 kPa confining pressure according to rubber volume content.

**Figure 22:**
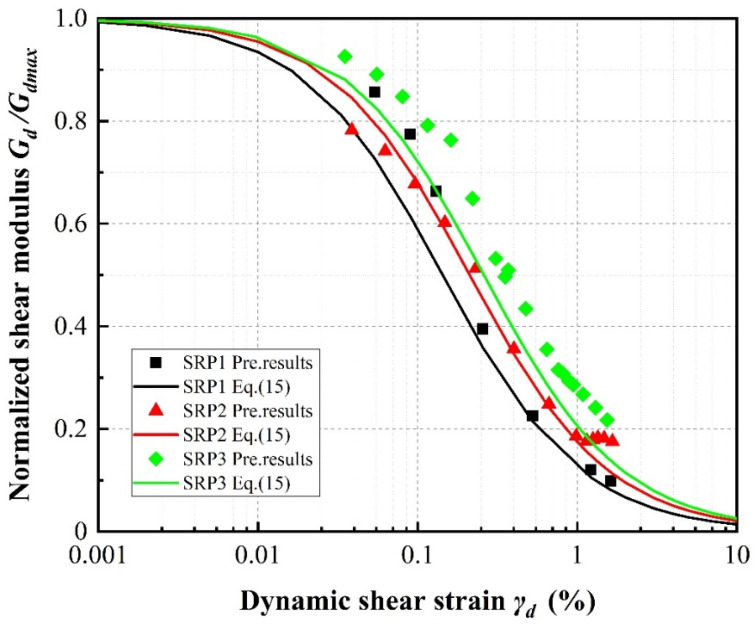
Normalized shear modulus versus shear strain for 20% rubber volume content under 100 kPa confining pressure according to rubber particle size.

**Figure 23:**
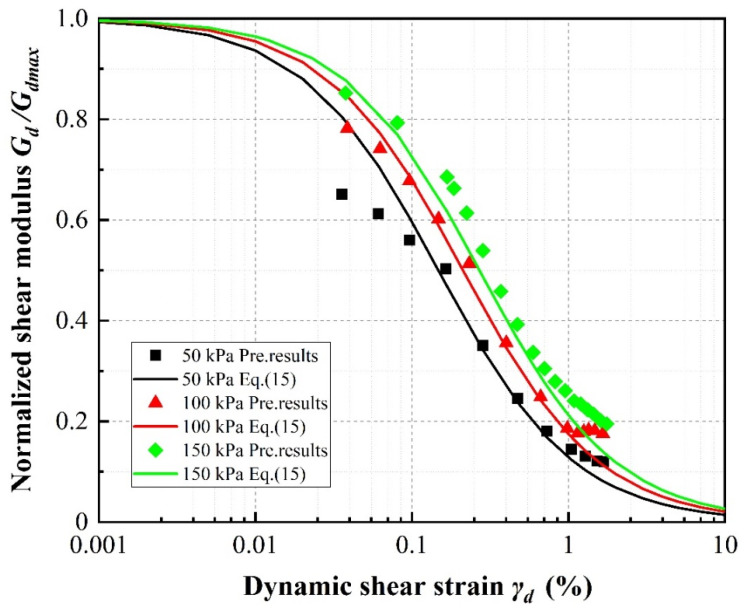
Normalized shear modulus versus shear strain amplitude for 20% rubber content SRP2 according to applied confining pressure.

Figure 21, Figure 22 and Figure 23 show the normalized dynamic shear modulus, defined as *G_d_*/*G_dmax_*, versus dynamic shear strain under various rubber volume content values, rubber particle sizes and confining pressures, respectively. The values of *G_d_*/*G_dmax_* shown in the curves were determined using the right side of Equation (15) and the predicted results were determined by applying the results of Equation (16) as *G_dmax_* on the left side. It can be observed that the predicted results are in relatively good agreement with the calculation results, further illustrating that the new model can be confidently used to predict the attenuation of the shear modulus in the design of sand–rubber mixtures. For any constant confining pressure and rubber particle size, increasing rubber volume content causes the values of *G_d_*/*G_dmax_* to increase. This phenomenon is the result of an increase in material stiffness with increasing confining pressure and indicates more elastic and flexible (uniform) mixture behavior with increasing rubber volume content (Figure 21). It can be further observed that the values of *G/G_dmax_* increase with increasing rubber particle size (Figure 22) and confining pressure (Figure 23).

## 7. Summary and Conclusions

In this study, a series of large-scale consolidated undrained cyclic triaxial tests were conducted and a systematic analysis of the results was undertaken to investigate the dynamic behavior of sand–rubber particle mixtures under a wide range of shear strain amplitudes. The impacts of various parameters including rubber volume content, rubber particle size, and confining pressure were considered in detail. These impacts were then incorporated into a proposed empirical model to predict the shear moduli of sand–rubber mixtures. The following conclusions are drawn from the test results and analysis:

1. Almost all samples exhibited strain-hardening behavior, that is, the maximum dynamic shear stress was observed at large strains. The stress–strain relationship under cyclic loading exhibited hysteresis, non-linearity, and strain accumulation. As the rubber volume content of the mixture specimens increased, the stress–strain curve shifted upward, whereas the results were the opposite as the confining pressure increased. The rubber particle size was also observed to affect the relationship between stress and strain.
2. As the rubber volume content increased, the shear moduli of all mixture specimens first decreased significantly but then slowly and finally tended to stability with ongoing increase in strain. However, increasing confining pressure and rubber particle size led to an increase in the shear modulus. This can be attributed to the number of sand–rubber and rubber–rubber contact points increasing with increasing rubber volume content, consequently decreasing the shear moduli of the mixture specimens.
3. The performance of the mixture specimen damping ratios was observed to be complicated. At shear strain amplitudes less than a critical strain value, the damping ratios of the mixture specimens increased with increasing rubber volume content and showed a higher damping ratio than pure sand. However, at shear strains higher than a critical strain value, the opposite trend was observed. In addition, the damping ratio decreased slightly with increasing confining pressure, and considerably increased with decreasing rubber particle size.
4. The maximum shear modulus was observed to decrease with increasing rubber volume content. The relationship between the maximum shear modulus and rubber volume content was found to be linear, while that between the maximum shear modulus and confining pressure was found to be exponential. An empirical model was, accordingly, proposed for the maximum shear modulus and verified against the experimental results. The verified empirical model was then used to calculate the reference shear strain and normalized shear modulus according to rubber volume content, rubber particle size, and confining pressure, as the attenuation of the shear modulus is critical to the accurate representation of the dynamic behavior of sand–rubber mixtures. It was found that when the confining pressure and rubber particle size were held constant, a higher rubber volume content resulted in higher normalized shear modulus (*G_d_*/*G_dmax_*) values. Furthermore, the normalized shear modulus values calculated using the experimental results were found to match the values predicted using the proposed empirical model. Thus, the proposed empirical model can serve as a reference for estimating the maximum shear modulus of a sand–rubber mixture.

The sand–rubber mixture has potential as a low cost and simple construction isolation material, although its dynamic behavior is very complicated. The purpose of this study was, therefore, to improve the current understanding of the dynamic behavior of these mixtures. The results of the experimental investigations indicate that applications of sand–rubber mixtures as isolation materials must carefully consider their engineering properties, as positive or negative effects can result from adjustments of the different parameters involved. The relative density may affect the dynamic behavior of the sand-rubber mixture, while this study is only considered for Dr = 50%. It is recommended that additional research is conducted to investigate the other effects including relative density, frequency and so on, between a rubber–sand mixture.

## Figures and Tables

**Table 1:** Main physical properties.

Type	Minimum Grain Size *D_min_* (mm)	Maximum Grain Size *D_max_* (mm)	Mean Grain Size *D*_50_ (mm)	Size Ratio
Sand	0.075	2	0.66	0
RP1	0.05	0.1	0.075	0.11
RP2	0.1	2	0.5	0.75
RP3	2	4	3	4.54

**Table 2:** Experimental conditions of cyclic triaxial test.

Type	Rubber Particle Size	Rubber Volume Content (%)	Size Ratio	Mean Effective Confining Pressure *σ*_3_ (kPa)	*ρ_dmax_* (g/cm^3^)	*ρ_dmin_* (g/cm^3^)	*D_r_*	*ρ_d_* (g/cm^3^)	Mixture Weight (g)
Pure Sand	-	0	0	50, 100, 150	1.97	1.72	0.5	1.84	158.6
SRP1	RP1	10	0.11	50, 100, 150	1.86	1.55	0.5	1.69	145.7
		20		50, 100, 150, 200	1.73	1.44		1.57	135.3
		30		50, 100, 150	1.61	1.31		1.44	124.1
		40		50, 100, 150	1.48	1.19		1.32	113.8
		50		50, 100, 150	1.29	1.07		1.19	102.6
SRP2	RP2	10	0.75	50, 100, 150	1.78	1.49	0.5	1.62	139.6
		20		50, 100, 150, 200	1.74	1.47		1.59	137.1
		30		50, 100, 150	1.67	1.31		1.47	126.7
		40		50, 100, 150	1.45	1.17		1.30	112.1
		50		50, 100, 150	1.33	1.07		1.19	102.6
SRP3	RP3	10	4.54	50, 100, 150	1.91	1.69	0.5	1.78	153.4
		20		50, 100, 150, 200	1.82	1.47		1.63	140.5
		30		50, 100, 150	1.75	1.43		1.57	135.3
		40		50, 100, 150	1.60	1.30		1.43	123.3
		50		50, 100, 150	1.47	1.17		1.30	112.0
